# An unexpected complication and an endovascular solution during endovascular repair of subclavian artery and thoracic aorta aneurysm

**DOI:** 10.1186/1749-8090-8-S1-O298

**Published:** 2013-09-11

**Authors:** L Cetin, M Canyigit, A Kucuker, M Hidiroglu, A Kunt, E Sener

**Affiliations:** 1 Ataturk Education and Research Hospital, Cardiovascular Department, Ankara, Turkey; 2 Ataturk Education and Research Hospital, Radiology Department, Ankara, Turkey

## Background

A variety of complications related to endovascular procedures are being reported. We present an annoying complication implying complete migration of stent-graft of subclavian artery into the aortic lumen.

## Methods

A 61 years old man with abdominal endovascular aortic stent-graft inserted previously, had a saccular aneurysm of descending aorta distal to left subclavian artery and a small dissection flap with saccular aneurysm originating from left subclavian artery. Since the patient refused to have any surgical procedure for left subclavian artery revascularisation, thoracic aortic-stenting for descending aorta just below subclavian origin and a second stent-graft for left subclavian artery just above the orifice was planned. Coverage of left subclavian artery was not preferred aiming to avoid possible cerebrovascular events. During intervention, stent-graft to be inserted inside subclavian artery migrated totally into aortic arch in a perpendicular fashion.

## Results

Bilateral femoral and left brachial artery accesses were done. A 16x41mm stent-graft was inserted inside left subclavian artery. While insertion with balloon dilatation, stent-graft migrated distally with the balloon forming a partial prolapse into aortic arch. We tried several endovascular maneuvers including inserting a second graft into subclavian artery to stabilize the first one or trying to press this graft with the one inserted into descending aorta, but unfortunately the stent totally migrated into aorta. We catheterized migrated stent with balloon catheter and were able to move the graft with inflated balloon fixing it. Stent-graft and inflated balloon were pulled slowly back together to proximal part of left arm of the previously inserted abdominal aortic stent-graft and stabilized it carefully. Control DSA showed settled graft without any stenosis.

## Conclusion

We present a bothering complication and an endovascular solution we performed without complication.

